# Effect of the cancer specific shorter form of human 6-phosphofructo-1-kinase on the metabolism of the yeast *Saccharomyces cerevisiae*

**DOI:** 10.1186/s12896-017-0362-5

**Published:** 2017-05-08

**Authors:** Darjan Andrejc, Alenka Možir, Matic Legiša

**Affiliations:** 1 Department of Synthetic Biology and Immunology, National Institute of Chemistry, Hjadrihova 19, Si-1000 Ljubljana, Slovenia; 2 Department of Polymer Chemistry and Technology, National Institute of Chemistry, Hajdrihova 19, Si-1000 Ljubljana, Slovenia; 3 Current address: Lek-Sandoz Company, Ljubljana, Slovenia

**Keywords:** Deregulated glycolysis, *Saccharomyces cerevisiae*, 6-phosphofructokinase, Cancer metabolism, Posttranslational modification

## Abstract

**Background:**

At first glance, there appears to be a high degree of similarity between the metabolism of yeast (the Crabtree effect) and human cancer cells (the Warburg effect). At the root of both effects is accelerated metabolic flow through glycolysis which leads to overflows of ethanol and lactic acid, respectively. It has been proposed that enhanced glycolytic flow in cancer cells is triggered by the altered kinetic characteristics of the key glycolytic regulatory enzyme 6-phosphofructo-1-kinase (Pfk). Through a posttranslational modification, highly active shorter Pfk-M fragments, which are resistant to feedback inhibition, are formed after the proteolytic cleavage of the C-terminus of the native human Pfk-M. Alternatively, enhanced glycolysis is triggered by optimal growth conditions in the yeast *Saccharomyces cerevisiae.*

**Results:**

To assess the deregulation of glycolysis in yeast cells, the sf*PFKM* gene encoding highly active human shorter Pfk-M fragments was introduced into *pfk*-null *S. cerevisiae*. No growth of the transformants with the sf*PFKM* gene was observed on glucose and fructose. Glucose even induced rapid deactivation of Pfk1 activities in such transformants. However, Pfk1 activities of the sf*PFKM* transformants were detected in maltose medium, but the growth in maltose was possible only after the addition of 10 mM of ethanol to the medium. Ethanol seemed to alleviate the severely unbalanced NADH/NADPH ratio in the sf*PFKM* cells. However, the transformants carrying modified Pfk-M enzymes grew faster than the transformants with the human native human Pfk-M enzyme in a narrow ecological niche with a low maltose concentration medium that was further improved by additional modifications. Interestingly, periodic extracellular accumulation of phenylacetaldehyde was detected during the growth of the strain with modified Pfk-M but not with the strain encoding the human native enzyme.

**Conclusions:**

Highly active cancer-specific shorter Pfk-M fragments appear to trigger several controlling mechanisms in the primary metabolism of yeast *S. cerevisiae* cells. These results suggest more complex metabolic regulation is present in *S. cerevisiae* as free living unicellular eukaryotic organisms in comparison to metazoan human cells. However, increased productivity under broader growth conditions may be achieved if more gene engineering is performed to reduce or omit several controlling mechanisms.

**Electronic supplementary material:**

The online version of this article (doi:10.1186/s12896-017-0362-5) contains supplementary material, which is available to authorized users.

## Background

The yeast *Saccharomyces cerevisiae* is one of the most important commercial microorganisms. In addition, it is the model organism most widely used to study the principles of molecular biology. Although baker’s yeast has the ability to use various carbohydrates, it grows best on fermentable sugars such as glucose or fructose. All sugars are catabolized through glycolysis to ethanol and carbon dioxide both aerobically and anaerobically. To increase bio-ethanol production, there were several attempts in the past to enhance glycolytsis in the yeast *S. cerevisiae*, mainly by overexpressing specific glycolytic genes. In one attempt, eight glycolytic enzymes were overexpressed, including those catalyzing irreversible steps in glycolysis, such as hexokinase, 6-phosphofructo-1-kinase, and pyruvate kinase. However, no effect on the ethanol formation rate was detected [1]. Similarly, another study showed that the increased expression of lower glycolytic enzymes did not result in a higher rate of ethanol production compared with the host strain. Only a transient 15 min increase in the fermentative capacity of a transformant was detected after the ATP demand was increased using glucose pulses in an aerobic chemostat [2, 3]. No increase of the glycolytic flux to ethanol has been observed with simultaneous overexpression of 6-phosphofructose-2-kinase (Pfk2) and 6-phosphofructo-1-kinase (Pfk1). Increased Pfk2 activity in cells has been shown to result in a higher level of fructose-2,6-bisphosphate (F2,6BP), which should act as a strong allosteric activator of Pfk1 [3]. However, most eukaryotic Pfk1 enzymes have also been reported to be inhibited by citrate and ATP, a mechanism that may antagonize F2,6BP activation [4]. No major effect on glucose consumption and ethanol production has been observed with a mutated Pfk that lacks allosteric control and is not inhibited by ATP [5]. However, overexpression of HXT1 or HXT7 hexose transporters that led to improvement in lactic acid excretion was observed with an engineered yeast strain [6] while glucose uptake and improved cell growth due to overexpression of the Gcr1 transcription factor were reported [7].

Enhanced glycolysis is at the root of the altered metabolism and rapid proliferation of cancer cells. It has been known for more than 90 years that tumors consume larger amounts of glucose compared to normal cells and convert the majority of the glucose into lactic acid [8]. This deviant energetic metabolism known as the “Warburg effect” has been included among the hallmarks of cancer [9]. Glycolytic fluxes in human cancer cells are largely comparable to those of rapidly proliferating endothelial cells, but they are much higher than those in various other non-proliferating human cells. In the human breast adenocarcinoma cell line MCF7 the glycolytic flux was found to be about 10-fold as strong as that in other healthy human cells [10]. It is important to realize that such differences in fluxes were observed during the growth of cell lines in media with identical composition.

In cancer cells, the pivotal factor in increased, deregulated glycolysis appears to be the posttranslational modification of 6-phosphofructo-1-kinase (Pfk1) [11]. Pfk1 is the key regulatory enzyme of glycolysis that catalyzes the phosphorylation of fructose-6-phosphate to fructose-1,6-bisphosphate (FBP) using Mg-ATP as a phosphoryl donor [12]. This enzyme is stimulated by F2,6BP, ADP/AMP, and ammonium ions, whereas citrate and ATP act as strong inhibitors [12]. During evolution, eukaryotic Pfk1 enzymes developed by duplication, tandem fusion, and the divergence of catalytic and effector binding sites of the prokaryotic ancestor [13]. However, the active site of eukaryotic enzymes is located only in the N-terminus portion while allosteric ligand binding sites that developed by mutation at the C-terminus enable fine-tuning of the regulatory enzyme.

In cancer cells, the human 85 kDa native muscle-type Pfk-M can be cleaved by a specific protease, forming a shorter fragment. The newly formed 47 kDa enzyme still possesses catalytic activity but has altered kinetic properties. This truncated enzyme is resistant to feedback inhibition by citrate and ATP, whereas some effectors, such as F2,6BP, increase the enzyme’s activity to a level higher than that of the native enzyme. Only the short 47 kDa fragments, and not the native 85 kDa Pfk-M, have been detected in four different tumorigenic cell lines with Western blot analyses, and similar fragments have also been detected in tumor tissues of mice after subcutaneous infection with tumorigenic B16-F10 cells [11]. Enhanced glycolysis is believed to cause metabolic and redox stresses in cancer cells and to be partly alleviated by lactate formation and excretion. These intracellular conditions also lead to increased levels of reactive oxygen species (ROS) in mitochondria [14].

At first glance, the metabolism of rapidly growing yeast cells and of cancer cells appears to be similar. Tumors are characterized by faster glucose consumption, with most of the glucose converted to lactate and excreted, despite an abundance of oxygen (the Warburg effect), while yeast cells growing at high specific growth rates accumulate ethanol under aerobic conditions (the Crabtree effect) [15]. However, in addition to some apparent similarities between the two effects, metabolic differences have been observed between the two types of cells [16].

The yeast *S. cerevisiae* as a free living unicellular microorganism has specialized to primarily metabolize fermentable sugars, like glucose and fructose. It is important to realize that free living microorganisms like yeasts are exposed to huge fluctuations of nutrients in nature and they have adapted their metabolisms from scarcity to abundance of nutrients. During the evolution several control mechanisms have developed to prevent unwanted side effects caused by fast fermentation like redox unbalancing and/or detrimental reactive oxygen species (ROS) formation. In contrast, the cells in multicellular organisms are normally exposed to a regular supply of nutrients that remain at relatively constant levels in the blood serum. Because there is no need to control detrimentally enhanced metabolic fluxes triggered by increased levels of nutrients, such controlling mechanisms may be lost during the evolution of metazoans.

In the present study, we demonstrate the influence of the highly active, citrate-resistant form of a modified human Pfk-M enzyme on yeast metabolism. A truncated human sf*PFKM* gene that enables the synthesis of active shorter fragments (sf) was introduced into a *pfk*-null yeast strain [17]. By constructing a yeast transformant that encodes shorter human Pfk-M fragments, we expected that the highly active modified Pfk-M enzyme would enhance glycolysis. However, some mechanisms that specifically counteract detrimental unrestricted glycolysis in yeast cells were perceived and are outlined in the paper. Ultimately, growth conditions were defined that enabled faster proliferation of yeast cells encoding the shorter human Pfk-M fragments than those with the native human Pfk-M enzyme as a sole Pfk1 form.

## Methods

### Strains, media, and growth conditions

The yeast strain HD56-5A (*MAT*α *ura3-52, leu2-3, 112his3-11, 15 MAL3 SUC2 GAL*) was used as a wild-type strain, and its isogenic *pfk*1, *pfk*2 null derivative, HD114-8D (*MAT*α *pfk1∷HIS3 pfk2∷HIS3 ura3-52, leu2-3, 112his3-11, 15 MAL3 SUC2 GAL*), was used as a recipient of recombinant human genes using p416 and p426 plasmids [18].

Human muscle-type Pfk-M cDNA (Clone ID2964710) was purchased from Geneservice Ltd. (www.geneservice.co.uk, Cambridge, UK). The human n*PFKM* gene encoding the native 85 kDa human Pfk-M and the truncated sf*PFKM* gene (h*PFKM*frg9 gene) encoding a 47 kDa shorter Pfk-M were prepared as previously reported [11]. Using a PCR technique, restriction sites were introduced at the 5′ (Xba I) and 3′ (BamHI) ends, which enabled cloning of the genes into the p416 plasmid. The primer 5′-GAATTATCTAGAGCCACCATGACCCATGAAGAGCACCATGC-3′ was used as a forward primer for both genes, 5′-TAATTCGGATCCTTACAGGCCCTCGAAACCATC-3′ as a reverse primer for the truncated gene, and 5′-TAATTCGGATCCTTAGACGGCCGCTTCCCC-3′ as the reverse primer for the native gene. The n*PFKM* and sf*PFKM* genes were transformed into the *pfk*-null HD114-8D strain using the p416 plasmid, whereas the wild-type HD56-5A strain was transformed using an empty p416 plasmid to complement *ura*3 auxotrophy. For the spot assay, HD56-5A strain was transformed also with the p416GPD-sf*PFKM* plasmid. As a negative control, the HD114-8D strain was transformed using an empty p416 plasmid.

Initially, all transformants were grown on supplemented minimal medium (SMM), comprising synthetic dropout medium without uracil (Sigma-Aldrich, St. Louis, MO, USA) containing 2% glycerol and 2% ethanol (SMM-GE) as non-fermentable carbon sources. As a nitrogen source, yeast nitrogen base without amino acids or ammonium sulfate (Sigma-Aldrich, St. Louis, MO, USA) was used, with the addition of glutamine (0.25 g/L).

SMM was also used for testing the growth characteristics of the transformants in the presence of other carbon sources (spot assay). For growth on plates, 1.5% agar was added to the medium. For the initial tests on SMM with the different carbon sources, cells were grown on glycerol/ethanol medium in a submerged culture to mid-exponential phase, harvested by centrifugation, washed three times with sterile water, and re-suspended at 10^7^ cells/mL. Then, 5 μL of concentrated suspensions and of four 10-fold dilutions were spotted on SMM agar plates with specified carbon sources.

To follow the growth kinetics under submerged conditions, the transformants were grown in 500 mL baffled Erlenmeyer flasks with 100 mL medium on a rotary shaker at 100 rpm and 30 °C. The media were inoculated with a single cell colony pre-grown on SMM-GE medium. Growth kinetics were monitored by measuring optical density (OD_600_) with a spectrophotometer (Lambda 25, Perkin-Elmer, Boston, MA, USA) and growth coefficients determined as described in the Supplementary material (Additional file 1: Figure S1).

The maltose, ethanol, acetate, and glycerol concentrations in the supernatants were measured using appropriate enzymatic kits (Megazyme, Bray, Ireland).

### Determining NADH/NADPH ratios

To obtain sufficient biomass, the cells were first grown in medium with no fermentative C source in the presence of glycerol and ethanol, until a value of 1 (OD_600_) was reached. Then, the cells were transferred to medium with 1% maltose (w/v) or maltose with ethanol (10 mM). They were harvested after 3 h of incubation in specified media. Cell free extracts for the measurements of NADH and NADPH levels, were prepared as described under the Supplementary material. NADH and NADPH levels were measured using an Amplite Fluorimetric NADH/NADPH Ratio Assay Kit. For the measurements of glycolytic intermediates the cells were grown in a 0,05% (w/v) maltose medium with added 10 mM ethanol and 10 μM FeSO_4_ until a value of 1 (OD_600_) was reached. After the extraction of metabolites as described under the Supplementary material, the amounts of metabolites were determined enzymatically as described previously [19].

### Gas chromatography–mass spectrometry (GC-MS)

GC-MS analysis was performed using a Hewlett Packard Agilent 6890 N gas chromatograph (Agilent Technologies) equipped with a mass spectrometer (Hewlett Packard Agilent 5973 N, Agilent Technologies). A 30 m length × 0.25 mm internal diameter capillary column (DB-35 ms, Agilent Technologies) with a 0.25 μm film thickness was used for separation. Helium was used as the carrier gas at a constant flow rate of 0.9 mL/min. The split ratio was 5:1, the split flow rate was 4.4 mL/min, and the injector volume was 5 μL. The oven temperature program was as follows: 50 °C (2 min) to 300 °C (5 min) at 30 °C/min. A calibration curve was generated for each analyzed compound using an authentic standard sourced from Sigma-Aldrich (St. Louis, MO, USA).

## Results

### Growth of transformants on different fermentative sugars

**Fig. 1:**
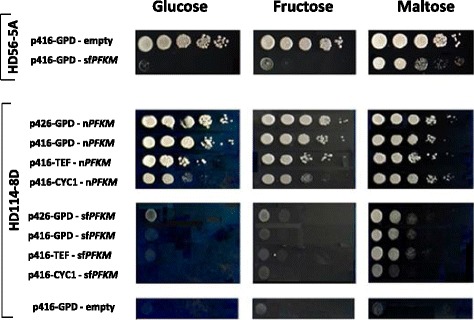
Strains carrying sf*PFKM* do not grow on glucose and fructose in a spot assay. Spot assay of the wild-type HD56-5A strain with empty p416 plasmid and p416-GPD plasmid with the inserted sf*PFKM* gene (*above*). In the second set (*below*), the growth of the *pfk* null HD114-8D strain transformants with the genes encoding native (n*PFKM*) and shorter fragments (sf*PFKM*) of human Pfk-M under the control of different constitutive promoters (*GPD, TEF*, and *CYC1*) is shown during the growth on SMM media with 1% of glucose, fructose or maltose

Initially, all of the transformants and the wild-type strain were tested for growth using a regular spot assay on SMM agar media, with glucose, fructose, or maltose as the sole carbon source. In this experiment, all sugars were added to the medium at a 1% w/v concentration. Surprisingly, only the wild-type strain and the transformant with the native human n*PFKM* gene grew well on the media with fermentative sugars, whereas the strain encoding the highly active shorter fragments displayed no growth on glucose or fructose and moderate growth on maltose during the incubation on solid medium. When the p416-GPD-sf*PFKM* plasmid was introduced into the wild-type HD56-5A strain, which contains the native yeast holoenzyme, a phenotype similar to the transformants with the sf*PFKM* gene was observed. It seemed that active shorter Pfk-M fragments negatively affected yeast metabolism and prevented the growth of all sf*PFKM* transformants during the cultivation on fast-fermenting sugars, glucose and fructose. Maltose enabled slower growth of the transformants compared to the wild-type and n*PFKM* strains but did not completely abolish cell proliferation (Fig. 1).

To obtain further information about the growth characteristics of the wild-type strain and transformants, the strains were further grown submerged in liquid SMM media with 1% (w/v) glucose, fructose, or maltose with or without ethanol (10 mM) in 500 mL baffled flasks on a rotary shaker.

### Expression of sfPFKM gene encoding short fragments of human Pfk-M and nPFKM gene encoding human native Pfk-M under the control of different promoters

To test whether the negative effects of the highly active shorter Pfk-M fragments on yeast metabolism could be reduced by decreasing the expression of the sf*PFKM* gene, constructs with three different constitutive promoters (*GPD*, *TEF*, *CYC1*) were prepared in a *pfk*-null yeast strain. Mumberg et al. [18] used a low-copy-number vector (CEN/ARS - p416) and showed that the β-galactosidase activity of the *lac*Z gene, which was expressed using the *TEF* promoter, was reduced by approximately fivefold, whereas under the control of the *CYC1* promoter, the expression was reduced by approximately hundredfold compared with that observed under the control of the *GPD* promoter. All tests were performed in a liquid 1% SMM maltose medium with ethanol added to the 10 mM concentration because better growth of sf*PFKM* transformants on maltose was observed if some ethanol was present in the medium. Minor differences were observed between the growth rate coefficients of the yeast transformants when the expression of the human sf*PFKM* as a sole Pfk1 isoform was controlled by either the *GPD* or *TEF* promoter. A slightly higher growth rate coefficient was observed with the *TEF* promoter (k′ = 0.1409) compared with that observed with the *GPD* promoter (k′ = 0.132); however, higher variability was observed among the parallels with the *TEF* promoter, with a calculated standard deviation of 0.228 (Additional file 2: Figure S2). Additionally, significantly slower growth was detected for the transformant with the *CYC1* promoter (k′ = 0.078), which was in the range of that of the transformant with the empty plasmid (Additional file 2: Figure S2). In contrast, no significant differences were observed among the growth rate coefficients of transformants that express the introduced n*PFKM* gene under control of the different promoters (Additional file 2: Figure S2).

The levels of the Pfk-M enzymes synthesized in the transformants carrying either of the two human *PFKM* genes under control of the different promoters were determined with immunoblotting. Although the levels of the native Pfk-M enzymes were reduced only slightly by decreasing the promoter strength, the shorter fragments were detected only when the genes were expressed by the *GPD* and *TEF* promoters. No protein was detected when the expression of the sf*PFKM* gene was under the control of the *CYC1* promoter (Additional file 3: Figure S3).

Thus, the transformant with the sf*PFKM* gene under the control of the *GPD* promoter was used for all further tests, because of the transformant’s superior reproducibility.

### Assay of Pfk1 activities

**Fig. 2:**
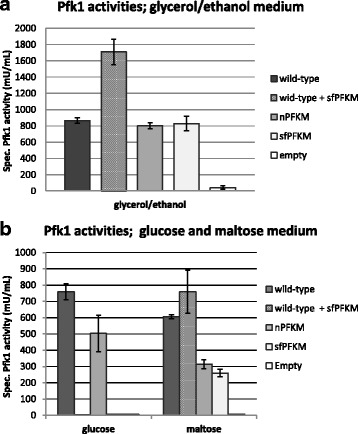
Glucose caused deactivation of PFK1 activities in the sfPFKM transformants. In glycerol/ethanol medium pre-grown cells Pfk1 specific activities were determined in the homogenates of the wild-type HD56-5A strain, n*PFKM* and sf*PFKM* transformants. No activity was observed in the host *pfk* null HD114-8D strain with the empty plasmid (**a**). However after the transfer of glycerol/ethanol pre-grown cells to the glucose medium Pfk1 activities were rapidly lost in the strains with sf*PFKM* genes (**b**). Data are present as means ± standard deviation

To verify whether active Pfk1 enzymes are formed in the wild-type strain and transformants, specific activities were assayed after the strains were grown in a medium with non-fermentable carbon sources (SMM-GE)(Fig. 2a) and after the transfer of the SMM-GE pre-grown cells into the SMM-glucose or SMM-maltose medium (Fig. 2b). Pfk1 activities were recorded in the homogenates of the wild-type strain and transformants carrying human *PFKM* genes grown on the SMM-GE medium (Fig. 2a). As expected, virtually no activity was detected in the *pfk-*null HD114-8D strain with the empty plasmid. These results proved that active recombinant Pfk1 enzymes were synthesized including the shorter Pfk-M fragments. However, significantly higher Pfk1 activities were recorded in the wild-type strain with the sf*PFKM* gene compared to the wild-type strain. The higher activity in the wild-type sf*PFKM* strain suggests that both wild-type yeast Pfk isoenzymes and human shorter fragments were active and contributed to the higher activity detected in the wild-type. Additionally, Pfk1 activities were measured after the transfer of the cells pre-grown in glycerol/ethanol (SMM-GE) medium into the 0.05% glucose or 0.05% maltose SMM medium. Interestingly, no activities were observed after 15 min of incubation of the transformants expressing the sf*PFKM* gene in the glucose medium while the activities of the wild-type and the n*PFKM* strain remained high (Fig. 2b). After the transfer of the cells into the 0.05% maltose SMM medium Pfk1 activities were recorded in cell-free extracts of all transformants. However, the activities of both strains with the inserted human *PFKM* genes as a sole *PFK* gene, were about two-fold lower than those of the strains with the wild-type yeast enzymes (Fig. 2b). Rapid decreases in Pfk1 activities were observed during the enzymatic measurements in the homogenates of all transformants with the sf*PFKM* genes. After approximately 2 min, activitiy was no longer detected in the buffer system. Therefore, only the activities detected immediately after the addition of the cell-free extract to the measuring system are presented in graphs. High initial activity followed by a rapid decrease was also detected in the wild-type strain transformed with the sf*PFKM* gene. This suggested that human shorter fragments likely interfere during the formation of the active Pfk complex among the native yeast subunits. The extreme instability of the shorter fragments under in vitro conditions, as observed and discussed previously [4], may be responsible for this rapid deactivation. It is important to realize that all measurements were conducted in a system without added enzyme inhibitors or activators to allow for a better comparison of different Pfk1 enzymes. In the assays a 1 mM ATP concentration was used that enabled optimal Pfk1 activities but was too low to induce inhibition. However, owing to the different kinetic characteristics of isoforms, the data presented in Fig. 2 do not reflect the intracellular activities.

### Submerged growth in 1% glucose, fructose, or maltose medium with or without ethanol

**Fig. 3:**
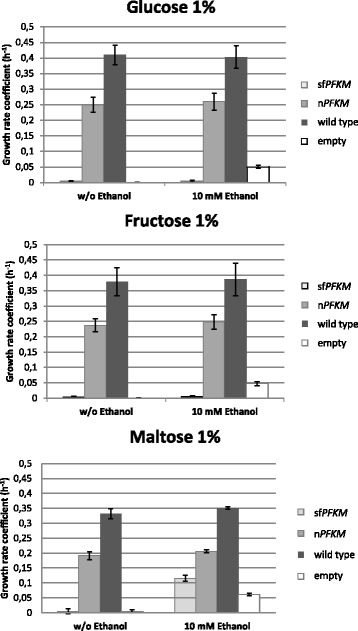
For growth of the sf*PFKM* strain on maltose, a low ethanol concentration was needed. Growth rate coefficients of the wild-type HD56-5A strain, host *pfk* null HD114-8DStrain (empty) and transformants with the native human (n*PFKM*) and modified human Pfk-M Enzymes (sf*PFKM*) on 1% glucose (**a**), fructose (**b**) or maltose (**c**) SMM medium without and with added ethanol (10 mM). Data are presented as means ± standard deviation

**Fig. 4:**
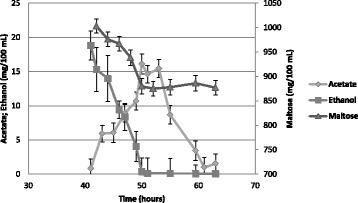
The growth of sf*PFKM* strain on maltose ceased after the ethanol was exhausted. Maltose (▲), ethanol (■) and acetate (♦) levels of the sf*PFKM* strain growing on 1% maltose SMM medium supplemented with 10 mM ethanol. Data are presented as means ± standard deviation

Similarly to the spot assay, the wild-type strain and the n*PFKM* strain with the native human Pfk-M grew well in submerged culture regardless of the sugar present. However, no growth of sf*PFKM* transformant was detected during the growth period in the liquid glucose or fructose SMM media with or without ethanol (Fig. 3). No growth of the sf*PFKM* strain was detected in the 1% maltose medium; however, the addition of 10 mM ethanol to the maltose medium positively affected the growth of the sf*PFKM* strain. Although the strain with the shorter Pfk-M fragments displayed relatively slow growth, with a k′ value of 0.115, it was nearly twofold faster than the growth rate of the HD114-8D *pfk*-null strain with the introduced empty p416 plasmid that reached a k′ value of only 0.061 (Fig. 3). Similar growth rate coefficients (k’ 0.063 ± 0.002) were detected with the wild-type, n*PFKM,* and sf*PFKM* strains growing on 10 mM ethanol as a single C-source. Measurements of maltose and ethanol consumption and acetate excretion in the transformant with the shorter Pfk-M fragments (Fig. 4) showed that growth proceeded only until all of the ethanol in the medium was consumed. From an initial concentration of 1 g per 100 mL, up to 0.85 g of maltose remained unused in the medium after the ethanol was exhausted. Accordingly, yeast cell growth stopped when the cell dry weight (CDW) reached approximately 0.6 g/L, whereas the yields of the wild-type strain and the strain with the native Pfk-M enzyme were 2.5 and 2.47 g/L, respectively. Characteristically, acetate was formed and excreted by the strain with the shorter Pfk-M fragments during ethanol consumption. The peak acetate concentration reached approximately 150 mg/L at the time of ethanol depletion and was consumed within approximately the next 10 h. In contrast, the wild-type strain and the transformant with the native human n*PFKM* gene neither consumed nor accumulated ethanol in the maltose medium with ethanol. However, when these strains were grown in medium with maltose as the single carbon source, ethanol was excreted, with up to 4 g/L produced by the wild-type strain and 0.8 g/L produced by the n*PFKM* transformant (the Crabtree effect). The growth rate coefficients of both strains were similar to those observed during growth on the maltose medium with ethanol.

### Pyridine nucleotide ratios

In the cells of all organisms, the balance between NADH and NADPH is of utmost importance. Both molecules function as freely diffusible electron carriers. NADH is involved in catabolic reactions such as respiration, while NADPH participates in anabolic reactions that consume energy to build or synthesize larger molecules. The correct ratio between the concentrations of both cofactors is therefore required for adequate electron fluxes between energy-releasing and energy-consuming processes that enable optimal cell growth. In the yeast *S. cerevisiae*, there is increased complexity in terms of balancing the formation and consumption of cofactors because there is no transhydrolase activity for converting NADH into NADPH [20].

**Fig. 5:**
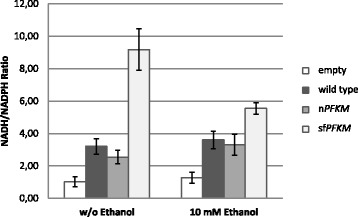
An unbalanced NADH/NADPH ratio is characteristic of the sf*PFKM* strain. The strains were pre-grown on glycerol/ethanol medium until the value of 1(OD_600_) was reached. As a control *pfk* null HD114-8D strain with empty plasmid has been taken. Pyridine nucleotide levels were measured 3 h after the transfer of the cells to 1% maltose or maltose with 10 mM ethanol medium. NADH/NADPH ratios were calculated from the data presented in Additional file 6: Table S1

However, ethanol consumption and acetate formation have been previously reported to play significant roles in balancing the NADH/NADPH ratio in yeast cells [21]. To determine whether ethanol acts as a redox balancer, we measured the NADH/NADPH ratios in the wild-type and in the n*PFKM* and sf*PFKM* transformants that grew on non-fermentable carbon source (glycerol/ethanol) after their biomasses were transferred into the maltose or maltose/ethanol medium (Fig. 5). In the fast-growing wild-type and n*PFKM* strain, the ratio between both pyridine nucleotides reached approximately 3:1 and 2.5:1 respectively in favor of NADH, whereas this ratio was only slightly increased in the presence of ethanol. An extremely high NADH/NADPH ratio was detected in the strain encoding the highly active shorter Pfk-M fragments, which was unable to grow in the medium with maltose as the sole C source, exhibiting a ratio of over 9:1. However, the presence of ethanol in the medium resulted in the reduction of the ratio to 5.5:1 (Fig. 5). Although the NADH level remained higher in the sf*PFKM* cells compared with that in the wild-type and n*PFKM* cells, the growth of the sf*PFKM* strain on maltose was re-established in the presence of ethanol.

### Differences in initial maltose concentrations

When the strains were grown in maltose/ethanol media with different initial sugar concentrations the highest growth rate of the wild-type and *nPFKM* strains was detected at 1% maltose and decreased with lower maltose concentrations. The peak growth rate coefficient of the sf*PFKM* strain was observed in the range of 0.05–0.1% maltose, whereas at higher maltose concentrations the growth coefficients gradually decreased (Additional file 4: Figure S4). At a 0.05% maltose concentration, the growth rate coefficients of the wild-type and n*PFKM* strain were slightly lower compared with that of the sf*PFKM* strain. Our final goal was to show that transformants with shorter Pfk-M fragments can grow faster than those with the human native Pfk-M enzymes. Therefore, we introduced further modifications to the 0.05% maltose/ethanol SMM medium to increase the difference between the growth rate coefficients of sf*PFKM* and n*PFKM* transformants.

### Effect of ferrous ions

**Fig. 6:**
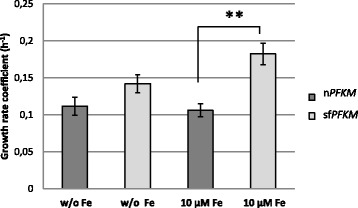
Ferrous ions accelerate growth of the sf*PFKM* strain but not of the n*PFKM* and wild-type. Growth rate coefficients of the sf*PFKM* and the n*PFKM* transformants on 0.05% maltose SMM medium with 10 mM ethanol and with or without 10 μM iron sulfate. Data are presented as means ± standard deviation. The significant (*p* <0.05, two-tailed *t*-test) increase in the growth coefficient of the sf*PFKM* compared with the n*PFKM* strain in the presence of ferrous ions is marked by (**)

We noticed that divalent iron ions increased the growth rate coefficient of the transformant encoding the shorter Pfk-M fragments but had no effect on that of the n*PFKM* strain. The addition of ferrous ions to the medium, in the form of 10 μM FeSO_4_, resulted in an increase in the growth coefficient of the sf*PFKM* strain from 0.142 to 0.182, whereas the growth of the strain with the native Pfk-M enzyme remained unchanged (Fig. 6). Overall, the growth of both strains in the medium with 10 μM FeSO_4_ added resulted in an approximately 50% higher growth rate coefficient for the strain with the shorter Pfk-M fragments compared with that of the strain with the native n*PFKM* gene.

Because ferrous ions were reported to stabilize the triose-phosphate isomeraze (Tpi) transcripts [22], the Tpi levels in transformants and wild-type strain were assessed by Western blot analysis. Results showed that a significantly lower amount of the enzyme was present in the sf*PFKM* strain than in the n*PFKM* and wild-type strains. Indeed, the addition of ferrous ions to the medium resulted in a distinct increase in the Tpi level in the sf*PFKM* strain but not in the n*PFKM* or the wild-type strain (Additional file 5: Figure S5).

### Levels of glycolytic intermediates

**Fig. 7:**
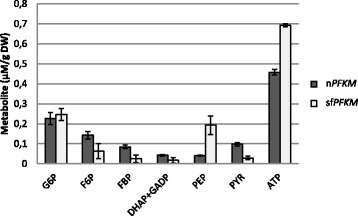
Levels of glycolytic intermediates. The leves of some glycolytic intermediates and ATP were measured in the sf*PFKM* and n*PFKM* strain growing in the medium that enabled faster growth of the sf*PFKM.* Abbreviations: *G6P* glucose-6-phosphate, *F6P* Fructose-6-phosphate, *FBP* fructose-1,6-bisphosphate, *DHAP*-dihydroxyacetone-3-phosphate, *GADP* glyceraldehyde-3-phosphate, *PEP* phosphoenolpyruvate, *PYR* pyruvate. The experiments were repeated three times and the data are presented as means ± standard deviation

In order to verify whether the activity of highly active shorter Pfk-M fragments had an effect on metabolism, profiles of glycolytic intermediates of both yeast transformants were determined and compared (Fig. 7). Both strains were grown in the 0,05% maltose medium with added ethanol (10 mM) and FeSO_4_ (10 uM) that enabled faster growth of the sf*PFKM* strain compared to the n*PFKM* strain. Glucose-6-phosphate (G6P) was present at the highest concentration amongst all glycolytic intermediates in both strains, however it was faster converted to fructose-6-phosphate (F6P) and fructose-1,6-bisphospte (F6P) in the sf*PFKM* strain. Another difference was observed at the level of phosphoenolpyruvate (PEP) where approximately 5 fold higher concentrations were detected in the sf*PFKM* strain. In contrast, pyruvate (PYR) levels were higher in the strain with the native Pfk-M inserted. ATP levels were measured as well. About 1.5 fold more ATP was present in the sf*PFKM* strain compared to the n*PFKM* strain. By determining glycolytic profiles by three independent experiments, higher standard deviations were obtained after the analyses of the results from the sf*PFKM* strain implying higher oscillations of glycolytic intermediates in this strain.

### Phenylacetaldehyde

**Fig. 8:**
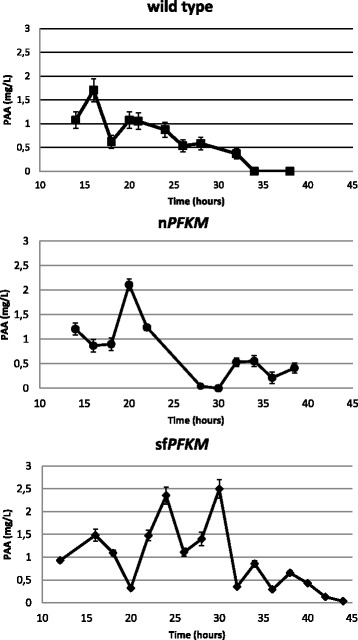
Peak phenylacetaldehyde concentrations reappear periodically only in the sf*PFKM* strain. Phenylacetaldehyde (PAA) levels of the wild-type strain (HD56-5A), the n*PFKM* and the sf*PFKM* strains detected in the 0.05% maltose SMM medium with added ethanol (10 mM) and Fe^2+^ ions (10 μM) by GC-MS. Representative examples of one out of three independent cultivations of each strain have been taken for GC-MS analyses. Data of three independent measurements are presented as means ± standard deviation

To determine whether the deregulated glycolytic flux in the sf*PFKM* transformants triggered a Crabtree-like effect, despite the relatively slow growth rate coefficient in 0.05% maltose medium, the extracellular metabolites in the medium were examined by gas chromatography. Surprisingly, phenylacetaldehyde (PAA) accumulation was observed, but no phenylethanol or phenylacetate was detected. The PAA concentrations quickly increased in the growth media of the all tested strains during the early hours of growth, reaching peaks between 1 and 2.2 mg/L when less than 0.1 g of dry cell weight per liter was present. The amount of PAA significantly decreased and remained low in the wild-type and n*PFKM* strain over the next 20 h. In contrast, another two peaks were observed at approximately 23 and 30 h of growth in the medium of the sf*PFKM* strain, with the peak values reaching 2.4 and 2.5 mg/L, respectively (Fig. 8).

## Discussion

In the present study, we showed that assumed deregulated glycolytic flux at the level of the Pfk1 enzyme was generated in the yeast cells by introducing human sf*PFKM* gene encoding cancer-specific, highly active, shorter Pfk-M fragments. However, increased glycolysis triggered several adverse effects in the yeast cells, which even prevented growth of the strain on fermentative sugars (Fig. 1). The most evident example of the detrimental results of the shorter Pfk-M fragments on metabolism was observed with the introduction of the sf*PFKM* gene into the wild-type yeast strain, where the addition of a single gene caused phenotypic changes similar to those of the sf*PFKM* strains. Surprisingly, we noted that a short exposure of the transformant with the highly active shorter Pfk-M fragments to glucose resulted in complete loss of Pfk1 activity while no such effect was observed with maltose in the medium (Fig. 2) The mechanism by which the shorter Pfk-M fragments were deactivated remains unknown, but it was very likely the result of a posttranslational modification such as phosphorylation. It has been reported that protein kinase A (Pka) can be activated very rapidly in yeast cells. Glucose addition to glucose-deprived cells triggered rapid activation (within 1 min) of the cAMP-PKA pathway which posttranslationally affected the activities of phosphatases, such as Glc7 and PP1 [23]. In budding yeasts glucose-induced cAMP signaling is sensed by extracellular glucose through the GPRC system, whereas the intracellular system depends on glucose uptake and hexokinase-mediated phosphorylation, which activates the Ras protein in some unknown way [24]. It may be that RAS proteins are activated by one or more glycolytic intermediates, where the concentration rapidly increases after the addition of glucose to the cells [25]. The increase of glycolytic intermediates may be even more severe in the sf*PFKM* strains encoding highly active, shorter Pfk-M fragments that are resistant to feed-back inhibition [11].

A negative effect of glucose on yeast metabolism has been recently also been described in the trehalose 6-phosphate synthase mutant (*tps*1Δ), which is deficient in the trehalose futile cycle. Growth arrest of the mutant after the dynamic change in the abundance of glucose has been described [26]. Such conditions were found to have the potential to imbalance the ATP-investing upper part and the ATP-producing lower part of glycolysis. It has been shown that phosphate dynamics are essential for the normal functioning of glycolytic flux. Specifically, the level of cytosolic phosphate (P_i_) was low at the onset of glucose addition to the mutant due to an unfunctional trehalose futile cycle when the ATP-demanding upper part of glycolysis was highly active. The decrease in P_i_ quickly became a limiting factor for the lower part of the pathway, thereby causing FBP accumulation [26]. This is more evidence of how tightly balanced the regulatory mechanisms of the primary carbon metabolism are in the yeast *S. cerevisiae*.

A less detrimental effect was observed during the submerged growth of the sf*PFKM* transformant on maltose/ethanol medium (Fig. 3). Maltose is a disaccharide known to allow slower yeast growth compared with that permitted by glucose. The primary reason for this effect has been reported to involve membrane transport mechanisms, with maltose entering cells via an energy-consuming proton symport mechanism, while glucose is transported by facilitated diffusion [27].

However, to learn more about the physiology of the sf*PFKM* strain, the maltose/ethanol medium composition first had to be further optimized for faster growth in liquid medium. Although the transformant encoding the native human Pfk-M enzyme showed similar physiological characteristics to those of the wild-type yeast strain, faster growth of the sf*PFKM* transformant compared with the n*PFKM* strain was possible only within a narrow ecological niche.

When the appropriate medium was chosen to allow the growth of both yeast strains with human *PFKM* genes, the expression levels of the introduced genes on growth were studied. The n*PFKM* and sf*PFKM* genes were expressed under the control of three different constitutive promoters of varying strengths in the recipient yeast cells. The native Pfk-M enzymes were formed and detected with immunoblotting, regardless of the promoter used, whereas the shorter fragments were produced only after expression of the sf*PFKM* gene under the strong *GPD* and moderate *TEF* promoters but not the *CYC1* promoter (Additional file 3: Figure S3). Recent studies of the three-dimensional crystal structures of mammalian Pfk1 [28, 29] have shown that the distal portion of the C-terminus is responsible for the binding of two dimers to form a tetrameric holoenzyme. Therefore, the shorter fragments, which were formed by the cleavage of the C-terminus, most likely assembled only in dimeric forms. Notably, active shorter Pfk-M fragments have been previously found to be extremely unstable. Under the diluted conditions, in the buffer during the kinetic measurements the monomers of holoenzyme were prone to rapid dissociation [11, 30] however, enzyme activities were found to be stable in a cell free extract, if protein concentration was higher than 5 mg/mL. Therefore, the formation of shorter Pfk-M fragments likely requires a higher intracellular concentration of monomers to bind and form active holoenzymes.

During growth on maltose/ethanol, the NADH/NADPH ratio was severely unbalanced in the sf*PFKM* transformants encoding highly active, shorter Pfk-M fragments (Fig. 5), primarily owing to the uncontrolled formation of glycolytic NADH. Because yeasts do not have nicotinamide dinucleotide transhydrogenase, which can convert NADH into NADPH [31], NADH began to accumulate. Still, the mitochondrion is the site of the most efficient re-oxidation of cytosolic NADH in *S. cerevisiae* cells growing aerobically. NADH dehydrogenases (Nde1 and Nde2) which are embedded in the inner mitochondrial membrane with their catalytic sites facing the intermembrane space, are involved in this process [32]. However, the rate-controlling step in the mitochondrial reoxidation of cytosolic NADH has been found to be the rate of diffusion of reduced nucleotides through the outer mitochondrial membrane and not the activity of NADH dehydrogenase [33]. It has been shown that the calculated permeability of NADH through the outer membranes of isolated yeast mitochondria is reduced at higher NADH concentrations [34].

The undesirable unbalanced nucleotide ratios were partially neutralized by adding ethanol to the maltose medium, which re-established the growth of the sf*PFKM* strain somewhat.. As shown in Fig. 4, ethanol consumption by the slow-growing sf*PFKM* strain was accompanied by extracellular acetate accumulation. Under high intracellular NADH levels, ethanol is probably first oxidized to acetaldehyde by strictly NADP^+^-specific alcohol dehydrogenase (Adh6). Although the major function of Adh6 is as an aldehyde reductase, some substrate specificity toward ethanol has been also detected [35]. Acetaldehyde can be than oxidized to acetate by cytosolic NADP^+^-specific acetaldehyde dehydrogenase (Ald6) which has also been reported to play an important role in balancing the NADH/NADPH ratios in yeast cells [21]. Therefore, conversion of 1 mole of ethanol to acetate would yield 2 moles of NADPH. The importance of ethanol for the metabolic processes of sf*PFKM* transformants is also demonstrated in Fig. 4, which shows that further maltose consumption and cell growth were halted after the depletion of ethanol from the medium.

Although similar growth rate coefficients were recorded among all three strains during growth on 0.05% maltose medium with added ethanol, we searched for additional differences between the growth rate coefficients of the sf*PFKM* and n*PFKM* strains. The addition of ferrous ions to the medium in the form of FeSO_4_resulted in a significant increase in the growth rate coefficient of the sf*PFKM* transformant but not that of the n*PFKM* strain. The stability of the triose-phosphate isomerase (*TPI1*) transcripts has been reported to be iron-regulated in *S. cerevisiae* [22] and to be present at levels at least three times higher in iron-supplemented media. Ferrous ions added at 10 μM to the medium led to a higher level of Tpi, especially in sf*PFKM* cells, in which a significantly lower amount of Tpi was detected with immunoblotting compared with the n*PFKM* and wild-type strains (Additional file 5: Figure S5). Therefore, it remains possible that an important, still unknown, control mechanism was acting to reduce the harmful unrestricted metabolic flow in the sf*PFKM* transformants at the level of Tpi.

Finally, a narrow ecological niche was determined enabling faster growth of the sf*PFKM* strain compared to the n*PFKM* strain. To test whether the activities of the Pfk-M shorter fragments could contribute to that, the levels of some glycolytic intermediates were determined. The data presented in Fig. 7 suggested faster conversion of the intermediates of the upper part of glycolysis in the sf*PFKM* that might be indeed caused by more active shorter Pfk-M fragments. Significantly increased NADH levels compared to those of NADPH (Fig. 5) and higher amounts of ATP (Fig. 7) might be explained by deregulated glycolytic flux at the level of Pfk-M as well. In addition, significant increase of PEP levels detected in the sf*PFKM* cells compared to the nPFKM cells might be another indication for accelerated glycolytic flux in the sf*PFKM* cells.

Pyruvate kinase (Pyk) was reported to play an important role in synchronizing redox metabolism in yeasts during respiration, which is characterized by low Pyk activity and phosphoenolpyruvate (PEP) accumulation in cells [36]. Moreover, PEP acts as a feedback inhibitor of Tpi that redirects some metabolic flow from glycolysis to the PP pathway and concomitantly enables NADPH formation [36]. The primary cause of PEP accumulation has been proposed to be the low activity of Pyk, which uses PEP as a substrate and is present in two isoforms in yeast cells. Pyk1 enzyme activity is stimulated by FBP [37] and Pka-mediated phosphorylation [38]; Pyk2 activity is not activated by FBP, and the gene expression of Pyk2 is subject to glucose repression [39]. Moreover, extremely low activity concomitant with a relatively high transcript level is characteristic of the Pyk2 enzyme [39]. During the growth of the yeast transformants on maltose medium in this study, the predominantly low Pyk2 enzymatic activity must have occurred because no glucose was present to trigger the formation of cAMP, which is required for Pka-dependent phosphorylation and the activation of Pyk1. Therefore, deregulated glycolytic flux at the level of Pfk1 may have caused increased PEP accumulation in the sf*PFKM* transformants.

High levels of G6P detected in both strains should enable some metabolic flow over the PP pathway, which is an important producer of NADPH. One of the intermediates of this pathway is erythrose-4-phosphate; together with PEP, this acts as a precursor for phenylpyruvate synthesis via the shikimate pathway in *S. cerevisiae* (http://www.genome.jp/kegg/pathway/see/see00400.html). Phenylacetaldehyde (PAA), which was found in the medium during the growth of the n*PFKM* and sf*PFKM* transformants, must have been formed from phenylpyruvate after decarboxylation. Normally, phenylpyruvate is transformed into PAA first and then reduced to phenylethanol (2-PE) by alcohol dehydrogenase (the Ehrlich pathway) [40, 41]. However, in the presence of ethanol in the medium, PAA, was detected, but 2-PE was not. Because peaks of PAA accumulation periodically recurred in the sf*PFKM* strain but appeared once and only transiently in the wild-type and n*PFKM* strains, a continuous undisturbed supply of precursors for the shikimate pathway must have been present in the sf*PFKM* strain, which may be explained by the undisturbed upper portion of the glycolytic flux. However, periodical appearance of PAA peaks (Fig. 8) as well as higher standard deviations of the levels of glycolytic intermediates measured in the sf*PFKM* strain (Fig. 7) imply strong oscillations of the glycolytic flux. It has previously been reported that in the yeast cells oscillations are driven by on/off switching of Pfk1 activity [42].

For all experiments only the batch fermentations were conducted so far, although chemostat-type continuous fermentations would be more appropriate and should be used for better assessment of growth and metabolic characteristics of the transformats with the inserted modified humen Pfk1 enzymes in the future.

## Conclusion

To conclude, the modified human sf*PFKM* gene enabled the synthesis of highly active shorter Pfk-M fragments in yeast *S. cerevisiae* cells, although the proliferation during growth on fast fermentable sugars was prevented by the deactivation of the Pfk1 enzyme, which occurred in an unknown way. However, slow growth of sf*PFKM* transformants was detected on maltose. By optimizing growth conditions on maltose, faster growth and better PAA production of the yeast transformants with modified Pfk-M compared with the cells with the human native Pfk-M enzymes were observed in a narrow ecological niche. Simultaneously, constraints inhibiting enhanced glycolysis were depicted.

Free living unicellular organisms such as yeast *S. cerevisiae* that are exposed to huge fluctuations of nutrients in the nature possess more complex controlling mechanisms that prevent unwanted side effects of enhanced metabolism compared to the human cells. By understanding these regulatory differences and by combining the specific genes from both types of eukaryotic cells, improved commercial organisms may be designed.

## Additional files

Additional file 1: Figure S1. Determination of growth coefficients. Growth rate coefficients were determined after the growth of the yeast cells was followed by measuring optical density of the medium (A). After the optical density values were converted to the dry weight using a calibration curve (B), maximum growth rates coefficients were calculated in the exponential growth phase (C). Data are presented as means ± standard deviation. (PDF 288 kb)

Additional file 2: Figure S2. Growth under the control of different promoters. Growth rate coefficients of transformants with different levels of sf*PFKM* and n*PFKM* gene expression were measured on liquid 1% maltose SMM medium with 10 mM ethanol. The genes were inserted into the transformants using the low-copy-number plasmid p416. Data are presented as means ± standard deviation. (PDF 340 kb)

Additional file 3: Figure S3. No shorter Pfk-M fragments were detected in the sf*PFKM* strain with low gene expression. Western blots of the *pfk* null host strain HD114-8D and transformants with the native and truncated versions of the human Pfk-M enzymes after expression under the control of different promoters (*GPD, TEF*, and *CYC1*). Glyceraldehyde-3-phosphate dehydrogenase (Gadph) has been taken as a loading control. (PDF 195 kb)

Additional file 4: Figure S4. The highest growth rates of sf*PFKM* strain were observed at low initial maltose concentrations. Growth rate coefficients of the wild-type strain (HD56-5A) and of the transformants on SMM media with different initial maltose concentrations and 10 mM ethanol. Data are presented as means ± standard deviation. (PDF 263 kb)

Additional file 5: Figure S5. Ferrous ions increase the levels of triose-phosphate isomerase (Tpi) in the sf*PFKM* strain. The amount of triose-phosphate isomerase (Tpi) determined by Western blot in transformants and wild-type strain with or without ferrous ions in the medium. Glyceraldehyde-3-phosphate dehydrogenase (Gadph) has been taken as a loading control. (PDF 187 kb)

Additional file 6: Table S1. Pyridine nucleotide levels detected in the wild type, n*PFKM* and sf*PFKM* strain. The strains were pre-grown on glycerol/ethanol medium until the value of 1(OD_600_) was reached. As a control *pfk* null HD114-8D strain with empty plasmid has been taken. Pyridine nucleotide levels were measured 3 h after the transfer of the cells to 1% maltose or maltose with 10 mM ethanol medium. In a table NADH, NAD^+^, NADPH and NADP^+^ levels from three independent measurements are shown. Data are presented as means ± standard deviation. (DOCX 39 kb)

## Abbreviations

Adh: Alcohol dehydrogenase
Ald: Acetaldehyde dehydrogenase
cAMP: Cyclic adenosine monophosphate
CDW: Cell dry weight
DHAP: Dihydroxyacetone-3-phosphate
F2,6BP: Fructose-2,6-bisphosphate
F6P: Fructose-6-phosphate
FBP: Fructose-1,6- bisphosphate
G6P: Glucose-6-phosphate
GADP: Glyceraldehyde-3-phosphate
GAPDH: Glyceraldehyde-3-phosphate dehydrogenase
HXT: Hexose transporter
n*PFKM*: Gene encoding native muscle type 6-phosphofructo-1-kinase
PAA: Phenyl-acetaldehyde
PEP: Phospho-enol-pyruvate
Pfk1: 6-phosphofructo-1-kinase
Pfk2: 6-phosphofructo-2-kinase
Pfk-M: Human 6-phosphofructo-1-kinase, muscle type*pfk –* gene encoding 6-phosphofructo-1-kinase
Pka: cAMP-dependent protein kinase
PP: Pentose phosphate pathway
Pyk: Pyruvate kinase
PYR: Pyruvate
ROS: Reactive oxygen species
sf*PFKM*: Gene encoding shorter fragments of 6-phosphofructo-1-kinase
SMM: Supplemented minimal medium
SMM-GE: Supplemented minimal medium with glycerol and ethanol
TCA: Tricarboxylic acid cycle
Tpi: Triose-phosphate isomerase
