# Supplementary figures and images for: Effect of the cancer specific shorter form of human 6-phosphofructo-1-kinase on the metabolism of the yeast Saccharomyces cerevisiae
Source: BMC Biotechnol. 2017 May 8;17:41. doi: 10.1186/s12896-017-0362-5 (PMC5422889; doi:10.1186/s12896-017-0362-5)

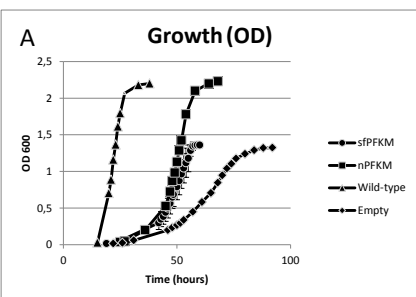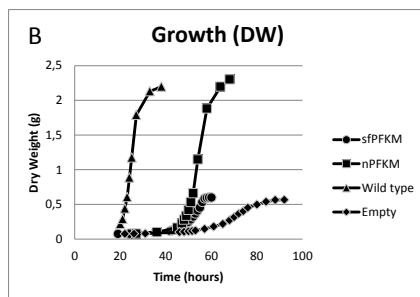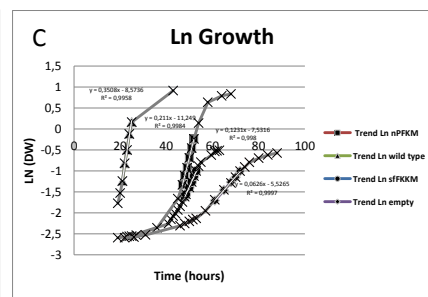

Supplement: Supplementary file 1 — Determination of growth coefficients. Growth rate coefficients were determined after the growth of the yeast cells was followed by measuring optical density of the medium (A). After the optical density values were converted to the dry weight using a calibration curve (B), maximum growth rates coefficients were calculated in the exponential growth phase (C). Data are presented as means ± standard deviation. (PDF 288 kb) [file 12896_2017_362_MOESM1_ESM.pdf]

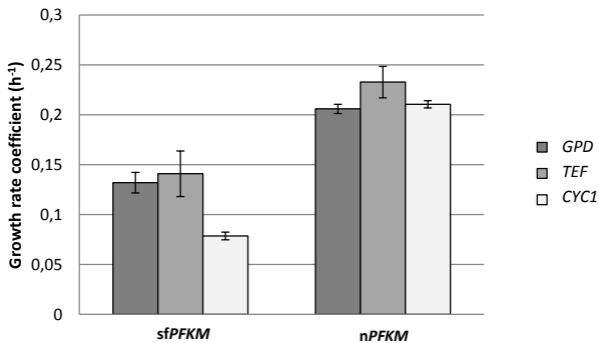

Supplement: Supplementary file 2 — Growth under the control of different promoters. Growth rate coefficients of transformants with different levels of sfPFKM and nPFKM gene expression were measured on liquid 1% maltose SMM medium with 10 mM ethanol. The genes were inserted into the transformants using the low-copy-number plasmid p416. Data are presented as means ± standard deviation. (PDF 340 kb) [file 12896_2017_362_MOESM2_ESM.pdf]

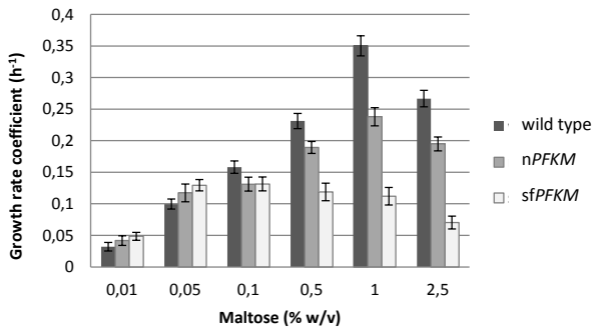

Supplement: Supplementary file 4 — The highest growth rates of sfPFKM strain were observed at low initial maltose concentrations. Growth rate coefficients of the wild-type strain (HD56-5A) and of the transformants on SMM media with different initial maltose concentrations and 10 mM ethanol. Data are presented as means ± standard deviation. (PDF 263 kb) [file 12896_2017_362_MOESM4_ESM.pdf]
