# Supplementary material for: Effect of the cancer specific shorter form of human 6-phosphofructo-1-kinase on the metabolism of the yeast Saccharomyces cerevisiae
Source: BMC Biotechnol. 2017 May 8;17:41. doi: 10.1186/s12896-017-0362-5 (PMC5422889; doi:10.1186/s12896-017-0362-5)
Supplement: Supplementary file 3 — No shorter Pfk-M fragments were detected in the sfPFKM strain with low gene expression. Western blots of the pfk null host strain HD114-8D and transformants with the native and truncated versions of the human Pfk-M enzymes after expression under the control of different promoters (GPD, TEF, and CYC1). Glyceraldehyde-3-phosphate dehydrogenase (Gadph) has been taken as a loading control. (PDF 195 kb) [file 12896_2017_362_MOESM3_ESM.pdf]

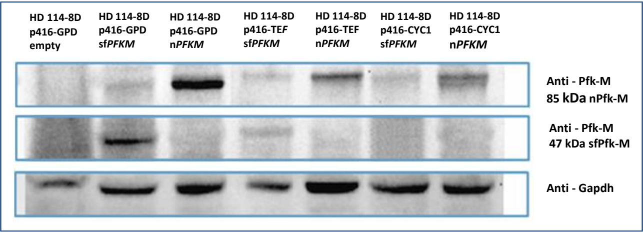
HD 114-8D p416-GPD empty
HD 114-8D p416-GPD sf*PFKM*
HD 114-8D p416-GPD n*PFKM*
HD 114-8D p416-TEF sf*PFKM*
HD 114-8D p416-TEF n*PFKM*
HD 114-8D p416-CYC1 sf*PFKM*
HD 114-8D p416-CYC1 n*PFKM*
Anti - Pfk-M
85 kDa nPfk-M
Anti - Pfk-M
47 kDa sfPfk-M
Anti - Gapdh
