# Supplementary material for: Effect of the cancer specific shorter form of human 6-phosphofructo-1-kinase on the metabolism of the yeast Saccharomyces cerevisiae
Source: BMC Biotechnol. 2017 May 8;17:41. doi: 10.1186/s12896-017-0362-5 (PMC5422889; doi:10.1186/s12896-017-0362-5)
Supplement: Supplementary file 5 — Ferrous ions increase the levels of triose-phosphate isomerase (Tpi) in the sfPFKM strain. The amount of triose-phosphate isomerase (Tpi) determined by Western blot in transformants and wild-type strain with or without ferrous ions in the medium. Glyceraldehyde-3-phosphate dehydrogenase (Gadph) has been taken as a loading control. (PDF 187 kb) [file 12896_2017_362_MOESM5_ESM.pdf]

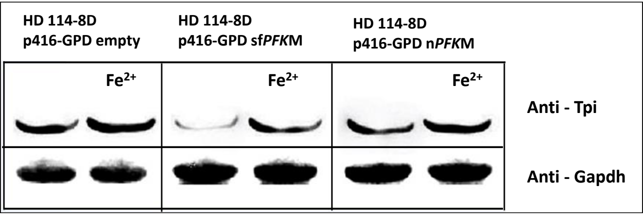
HD 114-8D
p416-GPD empty
HD 114-8D
p416-GPD sfPFKM
HD 114-8D
p416-GPD nPFKM
Fe2+
Fe2+
Fe2+
Anti - Tpi
Anti - Gapdh
