# Supplementary material for: Effect of the cancer specific shorter form of human 6-phosphofructo-1-kinase on the metabolism of the yeast Saccharomyces cerevisiae
Source: BMC Biotechnol. 2017 May 8;17:41. doi: 10.1186/s12896-017-0362-5 (PMC5422889; doi:10.1186/s12896-017-0362-5)
Supplement: Supplementary file 6 — Pyridine nucleotide levels detected in the wild type, nPFKM and sfPFKM strain. The strains were pre-grown on glycerol/ethanol medium until the value of 1(OD600) was reached. As a control pfk null HD114-8D strain with empty plasmid has been taken. Pyridine nucleotide levels were measured 3 h after the transfer of the cells to 1% maltose or maltose with 10 mM ethanol medium. In a table NADH, NAD+, NADPH and NADP+ levels from three independent measurements are shown. Data are presented as means ± standard deviation. (DOCX 39 kb) [file 12896_2017_362_MOESM6_ESM.docx]

**Supplementary material**

1. *Determination of growth coefficients*

Growth kinetics were monitored by measuring optical density (OD_600_) with a spectrophotometer (Lambda 25, Perkin-Elmer, Boston, MA, USA) (Fig. S1A). Dry weights (Fig. S1B) were determined from the optical density data using a calibration curve. For the calibration curve, dry weights were determined after cells were collected on dry, pre-weighted glass microfiber filters (GF/A), washed with deionized water and dried to a constant weight in an oven at 105°C. The growth rate coefficient (K’) was determined by the linear trend line prediction of the logarithmic values (Ln) of the dry cell weight increase during the exponential growth phase and expressed as reciprocal hours (h^-1^) (Fig. S1C) as reported previously [1]. Generation time (g) as a measure of time (h) can be obtained by the equation g = Ln2/K’. To illustrate, the three steps that were needed for the determination of growth coefficients of four different strains during one individual fermentation on 1% maltose/ethanol medium shown in Fig. 3 are presented in Fig. S1A, S1B and S1C. To determine mean values and standard deviation three parallel fermentations were conducted, OD values measured and growth coefficients determined,

1. *Expression of* sf*PFKM gene encoding short fragments of human Pfk-M and* n*PFKM gene encoding human native Pfk-M under the control of different promoters*

A low-copy-number centromeric (*CEN/ARS*) vector(p416) and *2 μ* high-copy-number (p426) [2] were obtained from ATCC (Manassas, VA, USA) and used for expressing the human native n*PFKM* gene and the truncated sf*PFKM* gene. Both plasmids contained the *URA3* marker gene to complement the *ura*3-52 auxotrophy of the recipient strains. In the p416 plasmid, the genes were under the control of constitutive promoters of varying strengths, including the glyceraldehyde-3-phosphate dehydrogenase [3] (*GPD*) promoter (ATCC 87360), translation elongation factor 1α [4] (*TEF*) promoter (ATCC 87368), and cytochrome-c [5] (*CYC1*) promoter (ATCC 87384). Only the *GPD* promoter was used to express the genes in the p426 plasmid (ATCC 87361).

1. *Cell-free extracts for the measuremenst of intracellular metabolites*

For the measurements of intracellular metabolites, including the NADH and NADPH levels, cell free extracts were prepared by collecting 50 mL of cells in 10 mL aliquots on a cellulose acetate filter with a pore size of 0.45 μm (Sartorius AG, Göttingen, Germany). After the cells in each aliquot were rapidly washed with ice-cold water, the membrane was placed in 2 mL of cold methanol (-20^o^C). The overall sampling of one aliquot was completed in less than 5 seconds. Tightly sealed Petri dishes containing methanol and immersed cells were sonicated for 30 seconds using a Transsonic 460 (Elma) sonicator. Finally, the methanol extract was placed into a chloroform (1.6 mL)/water (0.64 mL) mixture, extensively vortexed, and centrifuged at 2300 rcf for 5 min at 4°C. The upper aqueous phase was used for metabolite determination.

1. *Homogenate preparation and enzyme measurements*

For the measurement of specific Pfk1 activities, the strains were grown on SMM medium containing 2% glycerol and 2% ethanol. For the detection of the alcohol dehydrogenase activities, the cells were grown on 0.05% maltose SMM medium with added 10 mM ethanol and 10 μM ferrous ions. When OD_600_ reached a value of 1, the cells were harvested by centrifugation at 3000 rcf for 5 min and washed by centrifugation using ice-cold extraction buffer (50 mM HEPES buffer pH 7.5 containing 100 mM KCl, 1 mM DTT, 0.5 mM EDTA with added 10 μl of protease inhibitor cocktail (Sigma-Aldrich, Steinheim, Germany) per 100 mL of buffer). The cell pellet was frozen with liquid nitrogen and ground in the Mikro-Dismembrator (Sartorius AG, Göttingen, Germany). Proteins from the crushed cells were extracted with the appropriate amount of cold extraction buffer. After centrifuging at 12,000 rpm for 10 min in the refrigerated centrifuge (Sorvall, Wilmington, DE, USA), the homogenate contained about 5 mg of soluble proteins per ml. Protein concentrations in cell-free extracts were determined by protein assay (Bio-Rad, Hercules, CA, USA). Pfk1 activity was measured essentially as reported previously [6].

For the measurements of Pfk1 activities after incubation of the transformants in 0,05% glucose and 0,05% maltose medium, the cells were initially grown on 2% glycerol/ethanol SMM medium. When OD_600_ reached a value of 1, the cells were collected by centrifugation at 3000 rpm for 5 minutes, washed with 100 mM KCl by centrifugation and transferred into 0,05% glucose or maltose SMM medium. After 15 minutes the cells were collected by centrifugation, rinsed with the ice-cold extraction buffer and prepared for the measurements of Pfk1 activities.

Due to extreme instability of the shorter Pfk-M fragment’s activities under the diluted conditions [7], all reagents were first added to the buffer. Immediately after the addition of the cell free homogenate to the system the measurement of NADH consumption started. Only the initial, the highest enzyme activities were presented in the results. To obtain background activities of the homogenate, the measurements were performed in a system without added ATP.

1. *Immunoblotting*

For the Western blotting of Pfk-M, yeast cells were grown to an OD600 value of approximately 1.0. The cells were collected by centrifugation at 3000 rcf for 5 min, washed with cold distilled water, and frozen with liquid nitrogen. Then, they were disrupted using a Mikro-Dismembrator (Sartorius AG, Göttingen, Germany). The cell homogenates were separated by SDS-PAGE using 12% polyacrylamide gels with 0.1% sodium dodecyl sulfate after equal amounts of proteins were added to each well. Protein concentrations in cell-free extracts were determined by protein assay (Bio-Rad, Hercules, CA, USA). The membrane was blocked with blocking buffer, which was composed of 1x TBST and 5% w/v nonfat dry milk, and washed. For Pfk-M detection, the membrane was incubated with a 1:175 dilution of a purified primary antibody (polyclonal Pfk-M - antibody (T-18), 31710, Santa Cruz Biotechnologies, Santa Cruz, CA, USA) and subsequently incubated with a 1:1000 dilution of a secondary antibody (donkey anti-goat IgG-HRP, sc-2033, Santa Cruz Biotechnologies, Santa Cruz, CA, USA).

For triose-phosphate isomerase (Tpi) detection, the membrane was incubated with a 1:200 dilution of a rabbit primary antibody (http://www.antibodies-online.com/, ABIN459279), washed, and subsequently incubated with a 1:2000 dilution of a secondary antibody (goat polyclonal to rabbit IgG-HRP, ab6721, Abcam, Cambridge, UK). The membrane was developed with Amersham ECL Prime Western Blotting Detection Reagent (GE Life Sciences, Piscataway, NJ, USA), and luminescence was detected using a G:BOX (Syngene, Frederick, MD, USA).

As an internal control, glyceraldehyde3-phosphate dehydrogenase (Gapdh) was taken. The membranes were incubated with 1:2000 dilution of rabbit anti-Gapdh polyclonal antibody (<http://www.abcam.com/gapdh-antibody-loading-control-hrp-ab9385.html>). Identical secondary antibodies and the development procedure were used as for the TPI detection.

1. *Table*

Table S1 Pyridine nucleotide levels detected in the wild type, n*PFKM* and sf*PFKM* strain.

|  | **Medium w/o Ethanol** | |  | |  | |  | **Medium with 10 mM Ethanol** | | |  | |  |
| --- | --- | --- | --- | --- | --- | --- | --- | --- | --- | --- | --- | --- | --- |
|  | µM/gDW | | | | | | µM/gDW | | | | | | |
|  | **NADH** | **NAD^+^** | | **NADPH** | | **NADP^+^** | **NADH** | | **NAD^+^** | **NADPH** | | **NADP^+^** | |
| **wild type** | **0.370**±0.022 | **0.882**±0,025 | | **0.114**±0,021 | | **0.125**±0,015 | **0.327**±0,028 | | **1.138**±0,037 | **0.085**±0.007 | | **0.095**2±0,005 | |
| **n*PFKM*** | **0.324**±0,013 | **0.738**±0,02 | | **0.127**±0,018 | | **0.170**±0,028 | **0.336**±0,027 | | **0.961**±0.31 | **0.102**±0,01 | | **0.133**±0,012 | |
| **sf*PFKM*** | **0.702**±0,028 | **1.421**±0,016 | | **0.076**±0,005 | | **0.051**±0,004 | **0.288**±0,018 | | **1.021**±0,031 | **0.052**±0,003 | | **0.059**±0,011 | |

The strains were pre-grown on glycerol/ethanol medium until the value of 1(OD_600_) was reached. As a control *pfk* null HD114-8D strain with empty plasmid has been taken. Pyridine nucleotide levels were measured 3 hours after the transfer of the cells to 1 % maltose or maltose with 10 mM ethanol medium. In a table NADH, NAD^+^, NADPH and NADP^+^ levels from three independent measurements are shown. Data are presented as means ± standard deviation.

1. *Figures*

Fig. S1 Determination of growth coefficients

Growth rate coefficients were determined after the growth of the yeast cells was followed by measuring optical density of the medium (A). After the optical density values were converted to the dry weight using a calibration curve (B), maximum growth rates coefficients were calculated in the exponential growth phase (C). Data are presented as means± standard deviation.

Fig. S2 Growth under the control of different promoters

Growth rate coefficients of transformants with different levels of sf*PFKM* and n*PFKM* gene expression were measured on liquid 1% maltose SMM medium with 10 mM ethanol. The genes were inserted into the transformants using the low-copy-number plasmid p416. Data are presented as means ± standard deviation.

Fig. S3 No shorter Pfk-M fragments were detected in the sf*PFKM* strain with low gene expression

Western blots of the *pfk* null host strain HD114-8D and transformants with the native and truncated versions of the human Pfk-M enzymes after expression under the control of different promoters (*GPD, TEF*, and *CYC1*). Glyceraldehyde-3-phosphate dehydrogenase (Gadph) has been taken as a loading control.

Fig. S4 The highest growth rates of sf*PFKM* strain were observed at low initial maltose concentrations.

Growth rate coefficients of the wild-type strain (HD56-5A) and of the transformants on SMM media with different initial maltose concentrations and 10 mM ethanol. Data are presented as means ± standard deviation.

Fig. S5 Ferrous ions increase the levels of triose-phosphate isomerase (Tpi) in the sf*PFKM* strain

The amount of triose-phosphate isomerase (Tpi) determined by Western blot in transformants and wild-type strain with or without ferrous ions in the medium. Glyceraldehyde-3-phosphate dehydrogenase (Gadph) has been taken as a loading control.

*References:*

[1] Hagman A, Säll T, Compagno C, Piskur J. Yeast “Make-Accumulate-Consume” Life Strategy Evolved as a Multi-Step Process That Predates the Whole Genome Duplication. PLoS One 2013;8:e68734.

[2] Mumberg D, Müller R, Funk M. Yeast vectors for the controlled expression of heterologous proteins in different genetic backgrounds. Gene 1995;156:119–22.

[3] Bitter GA, Egan KM. Expression of heterologous genes in *Saccharomyces cerevisiae* from vectors utilizing the glyceraldehyde-3-phosphate dehydrogenase gene promoter. Gene 1984;32:263–74.

[4] Schirmaier F, Philippsen P. Identification of two genes coding for the translation elongation factor EF-1 alpha of *S. cerevisiae*. EMBO J 1984;3:3311–5.

[5] Guarente L, Lalonde B, Gifford P, Alani E. Distinctly regulated tandem upstream activation sites mediate catabolite repression of the CYC1 gene of *S. cerevisiae*. Cell 1984;36:503–11.

[6] Usenik A, Legiša M. Evolution of allosteric citrate binding sites on 6-phosphofructo-1-kinase. PLoS One 2010;5:e15447.

[7] Šmerc A, Sodja E, Legiša M. Posttranslational modification of 6-phosphofructo-1-kinase as an important feature of cancer metabolism. PLoS One 2011;6:e19645.
